# Biochemical Characterization of a Novel, Glucose-Tolerant β-Glucosidase from *Jiangella ureilytica* KC603, and Determination of Resveratrol Production Capacity from Polydatin

**DOI:** 10.1007/s12010-025-05272-7

**Published:** 2025-05-23

**Authors:** Arife Kaçıran, Miray Şahinkaya, Dilşat Nigar Çolak, Numan Saleh Zada, Murat Kaçağan, Halil İbrahim Güler, Hayrettin Saygın, Ali Osman Beldüz

**Affiliations:** 1 Department of Biology, Faculty of Sciences, Karadeniz Technical University, 61080 Trabzon, Turkey; 2 Department of Forestry, Dereli Vocational High School, Giresun University, Giresun, Turkey; 3 Department of Microbiology, Faculty of Biological Sciences, Quaid-I-Azam University, Islamabad, 45320 Pakistan; 4 Department of Molecular Biology and Genetics, Faculty of Sciences, Karadeniz Technical University, 61080 Trabzon, Turkey; 5 Department of Molecular Biology and Genetics, Faculty of Sciences and Arts, Ondokuz Mayis University, Samsun, Turkey

**Keywords:** *Jiangella ureilytica* KC603, β-glucosidase, Resveratrol, Glucose tolerant, Molecular Docking

## Abstract

Β-glucosidase, a ubiquitous enzyme, is responsible for catalyzing the hydrolysis of β-glycosidic linkages present in polysaccharides and contributes significantly to several industrial sectors such as food, agriculture, and biofuel production. β-glucosidases can convert polydatin to resveratrol through de-glycosylation. Resveratrol is important for human health and has potential applications in pharmacology. The preference of enzymatic conversion methods for resveratrol production improves the importance of β-glucosidases. The glucose tolerance of β-glucosidases also significantly impacts their applicability. Because the inhibition of many β-glucosidase’s activity by their reaction product, glucose, is a limiting factor for industrial applications. In this study, a robust β-glucosidase was isolated from a novel-defined *Jiangella ureilytica* KC603 strain. The β-glucosidase encoding gene (*JurBglKC603*) was cloned and expressed in *E. coli* BL21 (DE3) cells and a 50.1 kDa protein was purified using Ni-affinity column chromatography. The efficient polydatin deglycosylation capacity of JurBglKC603 was determined by Glucose Oxidase–Peroxidase (GOPOD) assay. JurBglKC603 exhibits remarkable resistance to glucose concentrations of up to 3 M. The enzyme remained active across a broad pH spectrum and was unaffected by most heavy metal ions, except for Hg^2+^. The kinetic parameters of JurBglKC603 were *K*_m_ = 0.44 mM, *V*_max_ = 26.87 U·mg^−1^, *k*_cat_ = 21.1 s^−1^, and *k*_cat_/*K*_m_ = 47,954 M^−1^·s^−1^ against *p*NPG and *K*_m_ = 4.6 mM, *V*_max_ = 20 U·mg^−1^, *k*_cat_ = 17.2 s^−1^, and *k*_cat_/*K*_m_ = 3822 M^−1^·s^−1^ against polydatin. Molecular docking studies have demonstrated that Gln19, His120, Trp411, and Glu410 play a vital role in the interaction with polydatin.

## Introduction

Polyphenolic compounds exist in nature and have significant importance in our daily lives. One such compound is resveratrol (trans-3,5,4 trihydroxystilbene), major component of grapes, berries, peanuts [1], and Chinese herb *Polygonum cuspidatum.* Resveratrol has significant value in the present era due to its therapeutic potential and can be used in the treatment of major diseases like cancer [2], cardiovascular diseases [3], pulmonary fibrosis [4], and age-related issues. The anti-inflammatory, antioxidant, and antimicrobial activities of resveratrol have been also reported [5]. Natural sources provide small amounts of resveratrol and its large-scale extraction is an expensive method, therefore, there is an increasing demand to develop alternative methods for obtaining resveratrol.

One possible method for resveratrol production is the extraction of resveratrol from its precursor form after modification [6, 7]. Polydatin (trans-piceid) is a polyphenolic compound and a glycosylated derivative of resveratrol, with a glucose moiety attached at the C-3 position. [8]. Polydatin is more abundantly present in plants compared to resveratrol but its glycosylated nature limits its potential. Moreover, its lower bioavailability makes it a less promising candidate for medicinal applications. Similarly, human intestinal cells have less affinity for the absorption of polydatin than resveratrol [7]. Conventional hydrolysis methods and chemical procedures are less suitable for the conversion of polydatin into resveratrol compared to enzymatic bioconversion. Hence, enzymatic de-glycosylation of polydatin is the most appropriate method for obtaining resveratrol [9].

The natural conversion of polydatin into resveratrol is carried out by the glycoside hydrolases (GHs) family [10]. In the hydrolysis of complex carbohydrates or sugars, the glycosidic bond is catalyzed by GHs family. Based on the nature of glycosidic linkages, type of sugar, and the aglycone moiety, hydrolysis of certain glycoside polyphenolic compounds is carried out by GHs [11, 12]. β -glucosidases (Bgls) are the most significant enzymes in GHs family and are capable of hydrolyzing β−1–4 glucosidic bonds in alkyl or aryl-β-D-glucosides, as well as in amino and cyanogenic glucosides, disaccharides, or oligosaccharides [13].

β-glucosidases exhibit a wide variety of applications in many industrial sectors such as ethanol production through the hydrolysis of cellulosic biomass, hydrolysis of isoflavone glycosides, catalytic machinery for the synthesis of alkyl glucosides, and processing flavor-enhancing agents in foods [14, 15]. Polydatin can be converted into resveratrol by β-glucosidase. The bioconversion of polydatin into resveratrol via direct enzymatic reaction is a more timesaving, economical, and efficient method than microbial whole-cell methods [11].

In a few research studies, resveratrol production from enzymatic hydrolysis was studied but the low expression level of β-glucosidase was a limiting factor of its incorporation into pilot-scale production. The step that limited the expression level was the feedback inhibition of the product, glucose. Therefore, novel, glucose-tolerant, highly efficient, and promising β-glucosidases are needed to meet industrial demands.

In this study, a newly identified, glucose-tolerant β-glucosidase (Bgl) from *Jiangella ureiltyca* KC603 (JurBglKC603) was identified, cloned, and biochemically characterized. The newly identified *Jiangella ureilytica* KC603, as reported by Saygin, H. et al. (2020), was selected for its notable activity in the esculin hydrolysis assay. The glucose tolerance of recombinant JurBglKC603 was assessed, and its potential for de-glycosylating polydatin to resveratrol was evaluated. Molecular docking simulations were conducted with the modeled structure of JurBglKC603 and polydatin to identify key amino acids involved in the de-glycosylation process. These findings provide a foundation for the rational design of JurBglKC603, enabling more efficient utilization of natural substrates, such as polydatin.

## Materials and Methods

### Substrate and Chemicals

A synthetic substrate, *para*-nitrophenyl β-D-glucopyranoside (*p*NPG), was acquired from Sigma-Aldrich (St. Louis, MO, USA). The Genomic DNA Extraction Kit, Plasmid DNA Isolation Kit, Taq DNA polymerase, restriction enzymes, T4 DNA ligase, and deoxynucleotide triphosphates were obtained from Promega (Madison, WI, USA). cOmplete™ His-Tag Purification Resin (Roche 5,893,801,001) was purchased from Merck. The chemicals were sourced from Sigma-Aldrich, and solutions were prepared using double-distilled and deionized water.

### Bacterial Strains, Vectors, and Media

*Jiangella ureilytica* KC603 was supplied by Hayrettin SAYGIN, Department of Molecular Biology and Genetics, Ondokuz Mayıs University, Samsun, Turkey. The pGEM-T Easy cloning vector (Promega, A1360) and *Escherichia coli* JM101 cells (NEB) were employed for cloning, and the pET28a(+) vector (Novagen) and *Escherichia coli* BL21 (DE3) cells (NEB) were utilized for recombinant protein expression. The ISP2 medium was utilized for the growth of *Jiangella ureilytica* KC603, while LB broth was used for cultivating *Escherichia coli* strains.

### Esculin Assay

*Jiangella ureilytica* KC603 was grown in ISP2 medium by incubating at 30 °C overnight. The β-glucosidase activity of *Jiangella ureilytica* KC603 by the esculin assay was determined as follows: a plate assay was performed using Luria–Bertani (LB) agar supplemented with 1% Esculin and 0.5 g/L ferric ammonium chloride. β-glucosidase acts on esculin to produce esculetin and glucose, which is reduced in the presence of iron and produces a black color at the hydrolysis site, indicating β-glucosidase activity [16].

### Amplification of *JurBglKC603* Gene

DNA isolation kit (Promega) was used to isolate genomic DNA from *Jiangella ureilytica KC603*. The strain was incubated in ISP2 liquid medium at 30 °C for 2–4 days, harvested by centrifugation, and stored. For pre-lysis of the cells, the pellet was dissolved in TE buffer (10 mM Tris–HCl, 1 mM EDTA, pH ~ 8) and stored at − 80 °C for 20 min, followed by heat shock at 100 °C for 5 min. A total of 120 µl of lysozyme (50 mM) was added, and incubation continued for 24 h at 37 °C, followed by genomic DNA isolation using the Promega genomic DNA isolation kit, following the manufacturer’s instructions.

The following primers were used for the amplification of the full-length JurBglKC603 gene, *BglKC603_F* (5′- CCCATg gCC TTC CgC TTC CCg −3′) as forward and BglKC603_RHis (5′- CTC GAG CGC GTC CTC ACT CTC C −3′) as reverse, having *Nco*I and *Xho*I restriction sites (underlined), respectively. PCR conditions were set as follows: a total of 35 cycles with 120 s of initial denaturation at 95 °C, 50 s of denaturation at 95 °C, 50 s of annealing at 49 °C, and 90 s of extension at 72 °C. The PCR product was visualized on 1% agarose gel on the BioRad Gel Imaging System.

### Cloning and Sequencing of *JurBglKC603* Gene

PCR products were inserted into the pGEM-T Easy cloning vector system for sequence analysis and subsequently confirmed by sequencing (Macrogen, Netherlands). GenBank Blast Program (NCBI, NIH, Washington DC) was used for similarity analysis. After confirmation, the *JurBglKC603* gene was cloned into pET28a (+) expression vector via *Nco*I and *Xho*I restriction sites and transformed into *E. coli* BL21 (DE3) host cells. The pET28a (+) containing a 1407 bp gene fragment was named pET-JurBglKC603 and used for intracellular expression of JurBglKC603 protein.

### Overexpression and Purification of *JurBglKC603*

The *E. coli* (DE3) cells with recombinant pET-JurBglKC603 vector were incubated overnight at 37 °C. The overnight culture was transferred to a new LB medium containing kanamycin (50 µg/mL) and grown at 37 °C until an optical density of 0.6 (OD_600_) was reached. The cells were induced with 1 mM isopropyl-β-D-thiogalactoside (IPTG) and incubated at 25 °C for an additional 16 h in a shaking incubator at 150 rpm. The induced cells were then collected by centrifugation at 10,000 rpm for 5 min and resuspended in phosphate buffer (100 mM, pH 7). Intracellular proteins were then released by sonicating the cells with Sartorius Labsonic M (80% amplitude, 0.6 cycles for 5 min) on ice, after centrifugation at 14,000 rpm for 15 min, the supernatant was stored for further analysis as crude protein extract.

His-tagged β-glucosidase was purified by Ni-affinity column chromatography. The column was equilibrated with an Equilibration Buffer (20 mM, pH 7.5 Tris–HCl, 200 mM NaCl, and 10 mM imidazole). The crude protein extract was loaded onto the column and washed with Washing Buffer (20 mM, pH 7.5 Tris–HCl, 200 mM NaCl, and 25 mM imidazole) to remove unattached proteins. β-glucosidase (JurBglKC603) was eluted from the column with an Elution Buffer (20 mM, pH 7.5 Tris–HCl, 200 mM NaCl, and 250 mM imidazole). The eluted fractions were analyzed by SDS-PAGE, and the concentration of purified proteins was determined by Bradford assay using bovine serum albumin as standard.

### Zymogram Analysis

A 12% Native-PAGE gel was prepared for the zymogram analysis of JurBglKC603, the purified enzyme was loaded onto the gel and run at 15–20 mA. Subsequently, the gel was stained using a solution containing 0.1% (w/v) esculin and 0.03% ferric chloride in a sodium phosphate buffer (100 mM, pH 8) [17]. The gel was then incubated at room temperature, and the activity of JurBglKC603 was observed as a dark band on a clear background.

### Enzyme Assay

To determine the activity of JurBglKC603 against *p*NPG substrates, 250 µL of the reaction solution, consisting of 50 mM sodium citrate buffer (pH 6), 0.8 µg of the enzyme, and 1 mM substrate, was incubated at 35 °C for 30 min. The reaction was stopped by adding 250 µL of NaHCO_3_ (50 mM) and the activity was determined by measuring the absorbance of the resulting colorimetric product (yellow) at 405 nm in a spectrophotometer [18].

### Effect of Temperature on the Activity and Stability of *JurBglKC603*

The impact of temperature on JurBglKC603 activity was evaluated using *p*NPG as the substrate under standard assay conditions, and the results were reported as relative activity (%). The optimum reaction temperature of the enzyme was determined by incubating the reaction mixture at different temperatures ranging from 20 to 55 °C. To assess the effect of temperature on the stability of JurBglKC603, the enzyme was incubated at different temperatures within the same range. The reactions were performed at specific time intervals with heat-incubated enzymes and measured spectrophotometrically. The reaction without heat treatment was used as control. All reactions were performed in triplicate.

### Effect of pH on Activity and Stability of *JurBglKC603*

To determine the effect of pH on the activity of JurBglKC603, the following buffers were prepared: 100 mM sodium citrate buffer (pH 3–6), 100 mM sodium phosphate buffer (pH 7–8), 100 mM glycine–NaOH buffer (pH 9–10), and 100 mM CAPS buffer (pH 11). The reactions were carried out at optimum temperature. For pH stability, JurBglKC603 was incubated in the buffers mentioned above at room temperature (the enzyme was stable at 25 °C). The reactions were performed at specific time intervals until the enzyme lost half of its activity. Results were reported in terms of residual activity against the *p*NPG substrate.

### Effect of Metal Ions, Detergents, Organic Solvents, and Other Chemical Reagents on *JurBglKC603* Activity

For evaluating the effect of metal ions on the activity of JurBglKC603 the following metals were used: Mg^2+^, Ni^2+^, Ca^2+^, K^+^, Zn^2+^, Cu^2+^, Mn^2+^, Hg^2+^, Na^2+^, Co^2+^, and Fe^2+^. The enzyme was incubated with 1 mM and 5 mM concentrations of each metal ion at 25 °C for 30 min. Following incubation, the reactions were performed at the enzyme’s optimum conditions.

The effects of detergents and organic solvents on the activity of JurBglKC603 were analyzed as follows: the enzyme was incubated with 1% and 5% concentrations of detergents (SDS, Triton X-100, Tween 20), 5% and 10% of organic solvents (DMSO, Dioxane, Methanol, Butanol, Ethanol, Isopropanol) and 1 mM and 5 mM concentrations of other chemical reagents (DTT, PMSF, DEPC, EDTA, EGTA) at 25 °C for 30 min. Residual activity was measured after 30 min of incubation at optimum pH and temperature using *p*NPG as the substrate. The activity of the untreated enzyme was considered 100%, and the remaining activity of the enzyme incubated with chemicals was calculated.

### Effect of Glucose on *JurBglKC603* Activity

Various concentrations of glucose (0, 25, 0, 5, 0, 75, 1, 2, and 3 M) were added to the reaction mixture to determine the effect of glucose on JurBglKC603 activity. The reactions were carried out under standard conditions using *p*NPG as the substrate.

### Enzyme (*JurBglKC603*) Kinetics

The kinetic parameters of JurBglKC603 were determined by performing serial reactions with *p*NPG substrate concentrations ranging from 0.1 to 7 mM, as determined from preliminary studies. The reactions were carried out with 50 mM sodium citrate buffer (pH 6) at 35 ℃ and measured at 405 nm. On the other hand, the extinction coefficient of *p*NPG for kinetic data calculations was determined at 405 nm. Michaelis–Menten constant (*K*_m_) and maximum velocity (*V*_max_) values were determined as the inverse of the values corresponding to the points where the *x-* and *y*-axes intersect in the Lineweaver—Burk plot [19].

The kinetic characteristics of JurBglKC603 against the polydatin substrate were also calculated using the GOPOD assay [20] based on the amount of glucose formed because of the reaction. JurBglKC603 was incubated at 40 °C for 1 h in sodium citrate buffer (50 mM, pH 6) with the addition of varying concentrations of polydatin (0.2–1 mM), afterward, the released glucose was measured with the D-Glucose (glucose oxidase/peroxidase; GOPOD) Assay Kit.

### TLC and DNS Analysis of Polydatin De-glycosylation

TLC (Thin Layer Chromatography) and DNS (3,5-dinitrosalicylic acid) analyses were also used to verify the effect of β-glucosidase on the polydatin substrate (Piceid). The reaction solution, consisting of 50 mM sodium citrate buffer (pH 6), 1 μg of enzyme, and 1 mM polydatin, was incubated at 40 °C for 1 h. After terminating the reaction, DNS and TLC analyses were performed.

The DNS test was performed to observe glucose production after treatment of polydatin with JurBglKC603. A 50 μL of the reaction product mentioned above was mixed with DNS solution and boiled for 10 min. The color change was measured spectrophotometrically at 540 nm.

TLC analysis was performed on pre-coated silica gel G plates. The separation of polydatin and resveratrol was achieved on TLC plates using ethyl acetate/hexane/glacial acetic acid (8:1:0.5, by volume) as the mobile phase. For product visualization, phosphomolybdic acid (dissolved in 10% ethanol v/v), which induces oxidation of both polydatin and resveratrol, was applied. The products appeared green after heating at 100 °C for 5 min.

### Homology Modelling and Molecular Docking Study for *JurBglKC603*

Molecular docking was performed to analyze the substrate binding affinity of JurBglKC603. The 3D structure of JurBglKC603, required for molecular docking, was obtained from SWISS-Model. The crystal structure of *Kribbella flavida* DSM 17836 (PDB ID: 6M6L) was used as a template to model the 3-dimensional structure of JurBglKC603 with an automated modelling program, SWISS-Model homology modular system (https://swissmodel.expasy.org). The predicted model quality was verified by Ramachandran plot, ERRAT, and 3D scores, the protein structure was analyzed with the PyMol molecular visualization software. The chemical structures of *p*NPG and glucose used for structural analysis of polydatin were downloaded from the PubChem database (https://pubchem.ncbi.nlm.nih.gov/). Potential polydatin and *p*NPG binding sites in the substrate channel were discovered by using AutoDock 4.2. The AutoDock program was employed to add polar hydrogens to the receptor molecule, and energy minimization was carried out by incorporating Kollman charges. The resulting data was saved in the PDBQT file format. Next, the substrate molecule (ligand) was prepared by assigning torsion angles, and it was also saved in PDBQT format. During the docking process, the receptor molecule was kept rigid, while the ligand remained flexible. A grid was generated to encapsulate the receptor (protein), and the lowest energy conformation was obtained by allowing the ligand to move within the grid. The docking results were obtained using AutoDock 4.2 software. To analyze the receptor-ligand complex, PyMol molecular visualization software (www.pymol.org) was utilized.

## Results and Discussions

### Bioinformatic Analysis of *JurBglKC603*

**Fig. 1:**
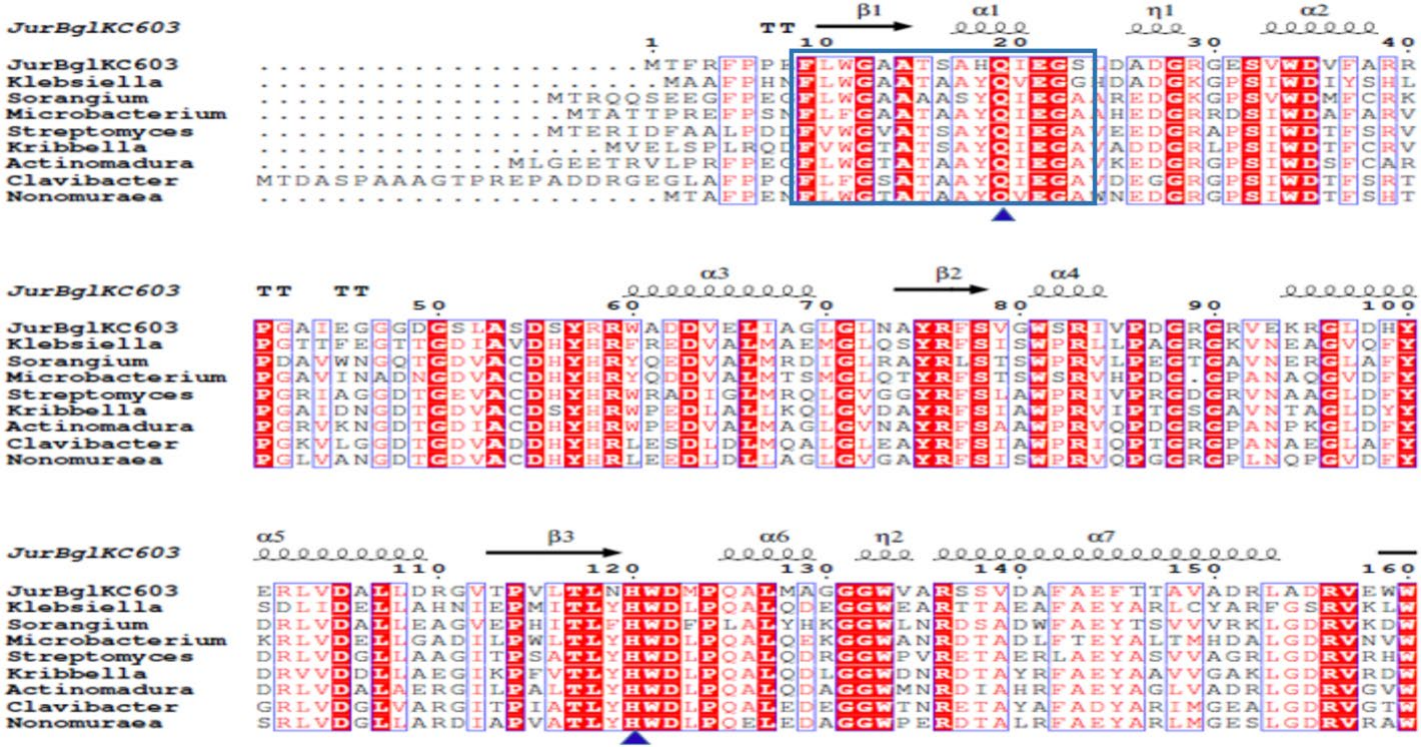

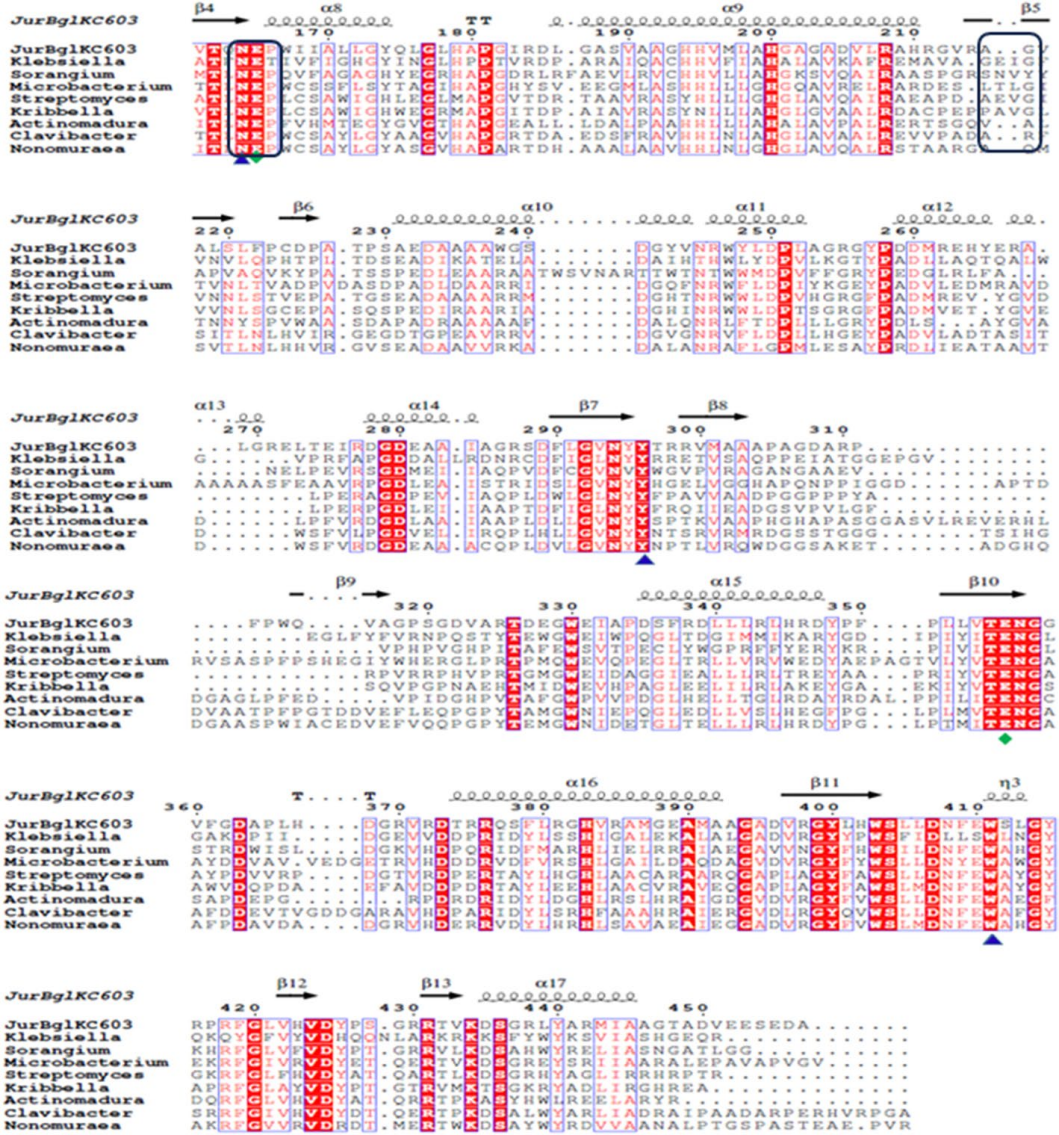
Sequence alignment of JurBglKC603 with homologous GH1 enzymes. The secondary structural elements of JurBglKC603 are shown at the top of the alignment. The blue triangle demonstrates conserved amino acids at the substrate binding site. Conserved protein donor and nucleophilic residues are indicated by the green tetragon. Regions enclosed in black boxes, NEPW (amino acids 164–167) and TENG (355–358) show characteristic conserved active site motifs. The region shown in the blue box represents the N-terminal signature sequence of JurBglKC603. Species names and GenBank numbers of β-glucosidase amino acid sequences are *Klebsiella pneumoniae* (QVR16087.1) (38, 41%), *Sorangium cellulosum* (CAN97832.1) (43%), *Microbacterium* sp. 2 FI (WP_137845323.1) (44%), *Streptomyces endophytica* (WP_265364230.1) (49%), *Kribbella flavida* (WP_012922836.1) (46%), *Actinomadura geliboluensis* (WP_138636094.1) (46%), *Clavibacter michiganensis* (WP_015489589.1) (43%), and *Nonomuraea zeae* (WP_138691944.1) (44%)

A ~ 1400 bp fragment was obtained through PCR amplification using the genomic DNA of *Jiangella ureilytica* KC603 as the template, confirming the presence of the amplified β-glucosidase gene (GenBank AN: TDC54154.1). The DNA sequence encoding JurBglKC603 was confirmed by Sanger Sequencing and the protein sequence, consisting of 457 amino acids, was determined by translating the gene sequence with Expasy software. The amino acid sequence of JurBglKC603 was aligned with other β-glucosidase sequences from UniProt and NCBI using Clustal Omega software, revealing two conserved active sites (Fig. 1). Using Expasy bioinformatics tools, the pI value of the JurBglKC603 was determined to be 5.35 and the theoretical molecular weight of the protein was calculated as 50.1 kDa (http://www.expasy.org/tools).

β-glucosidases are members of the glycoside hydrolases. A classification system for glycoside hydrolases, based on amino acid sequence and structural similarity, groups enzymes with similar sequences and conserved motifs into single families [14, 21]. Additionally, glycoside hydrolase families are further classified into superfamilies or clans based on similarities in catalytic domain structures, conserved catalytic amino acids, and tertiary structures [21]. GH1 belongs to clan GH-A (also known as superfamily 4/7), which is characterized by an eightfold α/β barrel structure [22]. Enzymes in this family, known for their broad substrate specificity and high sequence homology, possess a catalytic Glu (glutamic acid) region within TENG motifs, which functions as an acid/base catalyst, and another conserved Glu region within NEPW motifs, acting as an active catalytic nucleophile.

To identify the GH family of JurBglKC603, a BLAST search was performed using similar protein sequences from the NCBI database. The sequence similarities revealed that JurBglKC603 belongs to GH1 family. Further analysis of the amino acid sequencing using the UniProt database key features was identified, including the NEPW protein donor site, TENG nucleophile active site, and five substrate binding sites within conserved regions. These active and conserved regions were compared by aligning the sequence with GH1 family β-glucosidases from various bacterial species. JurBglKC603 contains a catalytic Glu (glutamic acid) at position 165, within the TENG motif, and another catalytic Glu at position 356, within the NEPW motif (Fig. 1). This data confirms that JurBglKC603 belongs to the glycosyl hydrolase I family.

GH1 family β-glucosidases possess a distinctive amino acid sequence at the N-terminus [23]. This sequence is characterized as (F-x-[FYWM]-[GSTA]-x-[GSTA]-x-GSTA-[FYNH]-[NQ]-x-E-x-[GSTA]). It is proposed that this N-terminal signature, critical for determining GH1 protein identity, may play a significant role in substrate recognition or the catalytic process [24]. The presence of the JurBglKC603 N-terminal signature sequence (FlWGaAtSAHQiEgS) was confirmed using ScanProsite (Expasy). The stability index of JurBglKC603 was calculated using the Expasy ProtParam tool (https://web.expasy.org/protparam), which estimates the in vivo stability of proteins. JurBglKC603 was classified as stable, with an instability index (II) of 36.13.

The aliphatic index (AI), a measure of the relative volume occupied by aliphatic side chains and an indicator of thermostability, was calculated as 79.30 for JurBglKC603, suggesting the protein is thermostable [25]. Protein solubility was assessed in vivo using the GRAVY (Grand Average of Hydropathy) value, calculated by summing the hydropathy values of all amino acids and dividing by the number of residues in the sequence. The GRAVY value of JurBglKC603 was − 0.300, with a negative value indicating that the protein is non-polar [26, 27].

### Cloning, Expression, and Purification of Recombinant *JurBglKC603*

The *JurBglKC603* gene was cloned into pET-28a (+) expression vector to create a recombinant plasmid and sequenced. After verifying the sequence accuracy by Sanger sequencing, pET-JurBglKC603 plasmid was transformed into *E. coli BL21* (DE3) cells and 16-h expression process was conducted at 25 °C. The recombinant protein was produced in large quantities as a soluble protein.

**Fig. 2:**
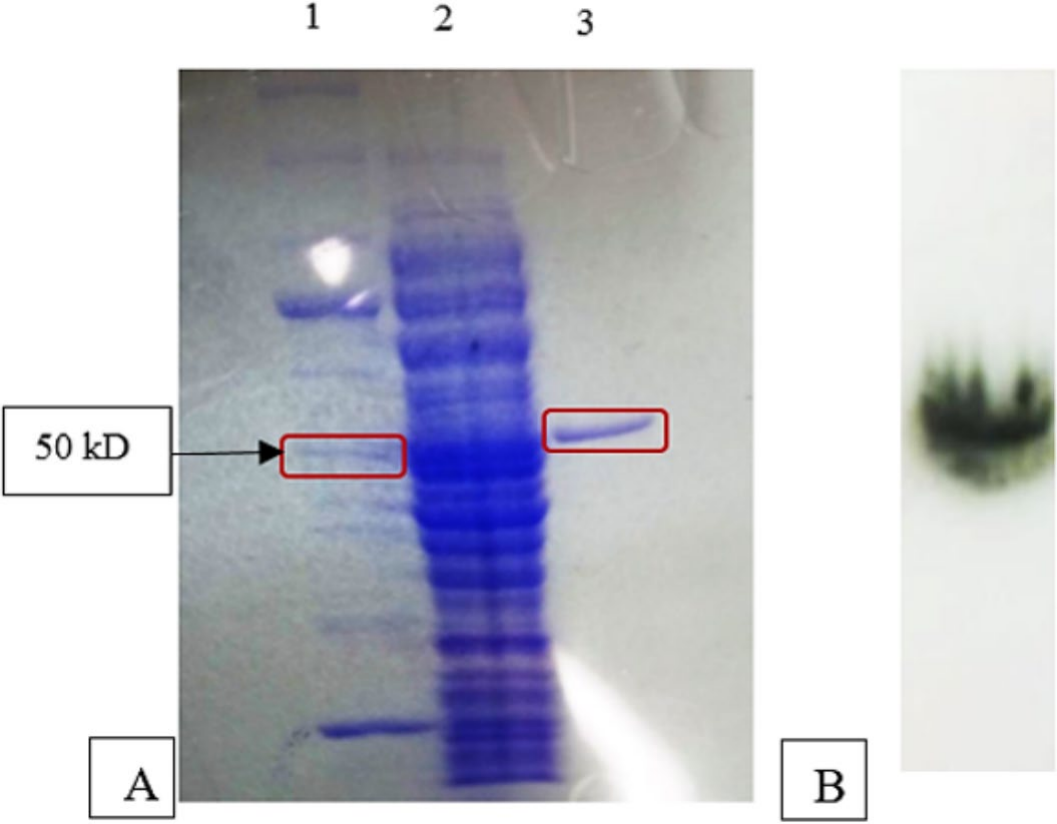
SDS-PAGE and Zymogram analysis of purified JurBglKC603 Protein. **A** Lane 1: Molecular weight marker (New England Biolabs Cat#P7719), Lane 2: Intracellular extract of *E. coli* BL21 (DE3) pLysS host cells harboring JurBglKC603 induced with IPTG, Lane 3: purified JurBglKC603 and **B** Zymogram analysis of purified JurBglKC603 protein*.* The black coloration against transparent background shows β-glucosidase activity on polyacrylamide gel electrophoresis under non-denaturing conditions

The purification of JurBglKC603, which carries a HisTag at the C terminus, was performed using Ni-affinity column chromatography and analyzed by SDS-PAGE. The molecular mass of the purified protein was determined as approximately 50 kDa through SDS-PAGE analysis (Fig. 2A). The activity of β-glucosidase was assessed post-purification using both the *p*NPG substrate and zymogram analysis. Hydrolysis of *p*-nitrophenyl-β-D-glucopyranoside (*p*NPG) by β-glucosidase, resulting in the formation of a yellow colorimetric product, confirmed β-glucosidase activity. Additionally, the formation of a black band against a transparent background in zymogram analysis further demonstrated β-glucosidase activity (Fig. 2B).

### Temperature and pH Properties of Recombinant Enzyme

**Fig. 3:**
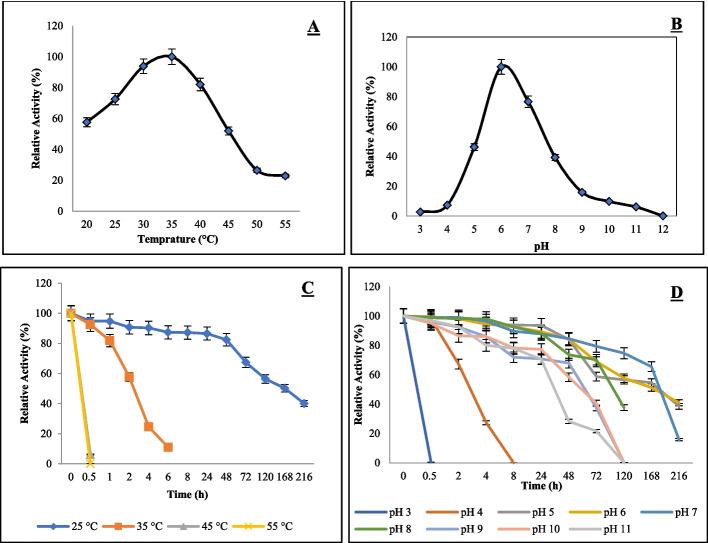
Graphical representation of temperature and pH optima and stabilities of JurBglKC603. **A** Optimum temperature of JurBglKC603, **B** optimum pH of JurBglKC603, **C** Thermal stability of JurBglKC603, and **D** pH stability of JurBglKC603

The enzyme exhibited an optimum temperature of 35 °C (Fig. 3). Temperature stability analyses revealed that JurBglKC603 maintained its activity at 25 °C for up to 24 h, after it began to decrease. However, the activity was completely lost after 6 h at 35 °C, and the enzyme retained its activity only for 30 min at temperatures ranging from 45 to 55 °C. Glycoside hydrolases active in low temperatures are predicted to reduce the time and cost of second-generation bioethanol production, and cold-active enzymes are preferred for heat-sensitive pharmaceutical and industrial applications [28]. Therefore, JurBglKC603 can be considered a suitable enzyme for industrial processes due to its stability and activity at low temperatures.

The enzyme exhibited an optimum pH of 6 (Fig. 3). pH stability analyses revealed that the enzyme lost half of its activity at pH 3 after 30 min and at pH 4 after 8 h. At pH 10–11, the enzyme’s half-life was 72 h. It retained half of its activity at pH 8–9 until the 5 th day, and at pH 5–7, activity was preserved for up to 216 h.

### Effect of Metals, Detergents, Organic Solvents, and Some Chemical Reagents on the Activity of *JurBglKC603*

**Fig. 4:**
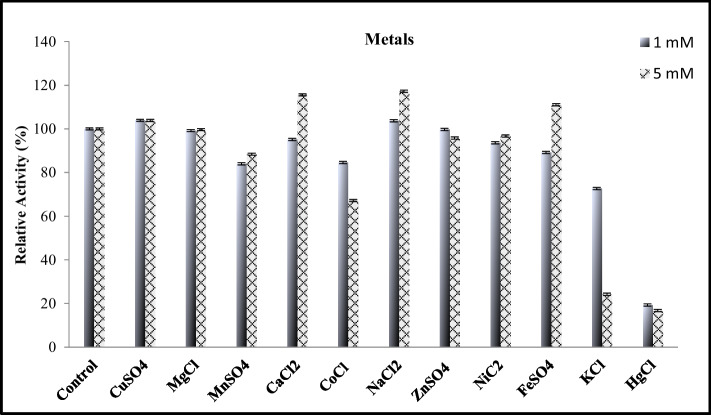
Effect of metal ions on JurBglKC603 activity

Β-glucosidase is a metal-independent enzyme and does not require metal ions for activity [17]. In this study, the effect of various metal salts (1 and 5 mM) on JurBglKC603 activity was investigated (Fig. 4). Consistent with the literature, heavy metals, except HgCl_2_, did not show a significant effect on the activity of JurBglKC603. The inhibitory effect of HgCl_2_ is likely due to the presence of cysteine residues, where the hydrogen ion of the sulfhydryl group is replaced by a covalent bond with the sulfur atom, resulting in enzyme inactivation [29, 30]. The metal tolerance of JurBglKC603 highlights its potential for use in various industrial applications [31].

**Fig. 5:**
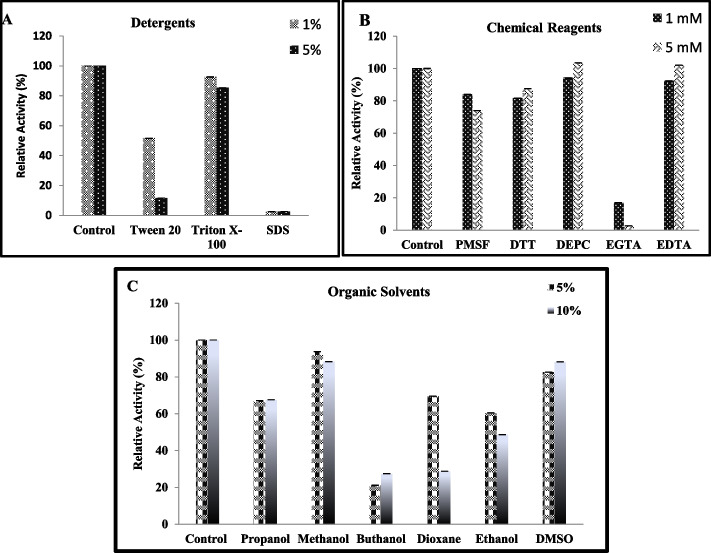
Effects of detergents, organic solvents, and inhibitors on JurBglKC603. **A** The effect of detergents on the β-glucosidase. **B** The effect of chemical reagents (inhibitors and chelators) on the β-glucosidase. **C** The effect of organic solvents on the β-glucosidase

Detergents such as SDS, Tween 20 and Triton X-100 were found to inhibit JurBglKC603 activity (Fig. 5A). While 49% of the enzyme activity remained in the presence of 5% Tween 20, it reduced to 8% in the presence of %1 Triton X-100. Enzyme activity was completely lost in the presence of 1–5% SDS. This inhibition may be attributed to SDS being a strong anionic detergent which interacts with the hydrophobic regions of the enzyme, leading to the unfolding of its native structure and subsequent denaturation [32].

The effects of the chelating agents EDTA and EGDA on the JurBglKC603 activity were markedly different. EDTA, a general chelating agent, slightly increased enzyme activity by 2%, whereas EGTA, a calcium-chelating agent, decreased activity by 98% (Fig. 5B). Both inhibitors are chelating agents with similar structures and functions; the difference is that EGTA’s affinity for the Ca^2+^ ions is higher than EDTA. The reason for the higher inhibition effect of EGTA may be due to the presence of Ca^2+^ ions at the active site of JurBglKC603 [33]. A 20% increase in enzymatic activity observed in the reaction with 5% Ca^2^⁺ further supports this finding (Fig. 4). GH1 (Glycoside Hydrolase family 1) β-glucosidases typically do not have a conserved calcium (Ca^2^⁺) binding site as a characteristic feature. Unlike some other glycoside hydrolases, which may rely on metal ions like calcium for structural stability or catalytic function, GH1 enzymes are often metal-independent [34]. However, there are exceptions depending on the specific enzyme or organism [24]. For instance, a Ca^2^⁺ binding site in the GH1 β-glucosidase derived from *Thermotoga maritima* was identified through X-ray crystallography. However, studies in the literature on the Ca^2^⁺ binding pocket of GH1-type β-glucosidases are limited [35]. However, the influence of calcium and EGTA on JurBglKC603 activity indicates the potential presence of a calcium-binding site within the enzyme.

Based on the theoretical understanding and experimental data, it is concluded that the presence of organic solvents significantly affects the activity of β-glucosidase (JurBglKC603) in a concentration- and hydrophobicity-dependent manner. Specifically, solvents with higher hydrophobicity, such as butanol, ethanol, and propanol, caused substantial reductions in enzyme activity, particularly at higher concentrations (10% v/v). At 5% concentration, butanol caused the largest decrease (79%), and at 10%, the reduction was even more pronounced (83%). In contrast, more polar solvents such as methanol and DMSO exhibited relatively lower inhibition, with methanol having the least effect (7% reduction at 5% v/v, 9% reduction at 10%).

The observed decrease in activity can be attributed to the alteration of the enzyme’s structure and function by the solvents. Organic solvents, particularly hydrophobic ones, disrupt the enzyme’s hydrophilic-hydrophobic balance, affecting its structural integrity and catalytic capacity. Since β-glucosidase requires water for hydrolysis reactions, the introduction of hydrophobic solvents reduces the availability of water molecules around the enzyme, impairing its ability to effectively catalyze the hydrolysis of glycosidic bonds. This leads to a decrease in enzymatic activity.

These findings suggest that while JurBglKC603 retains a degree of stability in the presence of less hydrophobic solvents, such as methanol and DMSO, its activity is significantly hindered by more hydrophobic solvents like butanol and ethanol. Therefore, for industrial applications involving organic solvents, particularly where the solvent presence is unavoidable, β-glucosidase activity may be compromised, and its use should be carefully optimized to minimize solvent-related inhibition.

### Glucose Tolerance

**Fig. 6:**
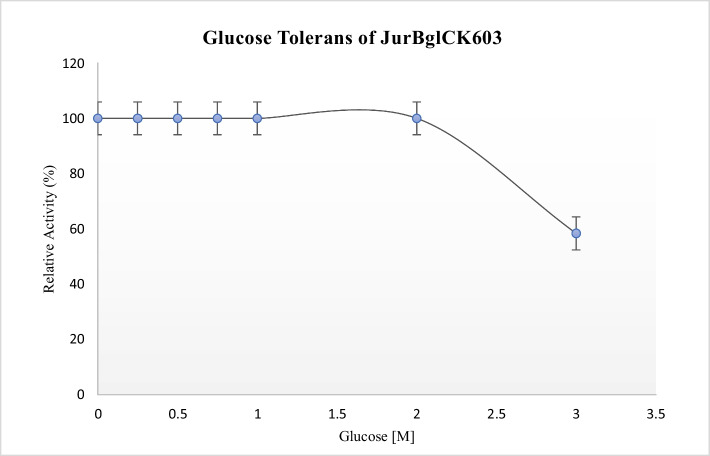
The glucose tolerance of JurBglKC603

JurBglKC603 activity was assessed using 4 mM pNPG, a substrate concentration at which the enzyme was saturated, in the presence of varying glucose concentrations (0.25, 0.5, 0.75, 1, 2, and 3 M). Relative activity was calculated by designating the activity of JurBglKC603 in the absence of glucose as 100%. The results indicated that JurBglKC603 retained activity in the presence of glucose concentrations up to 3 M (Fig. 6).

Although the exact mechanism underlying the inhibitory effect of glucose on Bgl activity remains unclear, it is well established that Bgl activity often decreases with increasing glucose concentrations. Amino acid residues located in the aglycone binding sites of β-glucosidases are thought to play a significant role in glucose tolerance [36]. According to Giuseppe et al., β-glucosidases with low glucose tolerance typically have a larger entrance to the active site tunnel and an electronegative patch along the tunnel. In contrast, those with high glucose tolerance have a narrower entrance, shorter tunnel length, and a gate controller region [37] The activity of JurBglKC603 showed no significant change with increasing glucose concentrations, retaining half of its activity even in the presence of 3 M glucose. Another β-glucosidase, Bgl A, derived from *Thermotoga naphthophila* RKU-10^ T^ is known as one of the enzymes tolerant to glucose [38]. Table 1 presents various β-glucosidases and their glucose tolerance levels as documented in the literature. JurBglKC603 stands out for maintaining high activity even at glucose concentrations up to 10 M, making it particularly advantageous for industrial applications.

**Table 1:** Glucose tolerances of various bacterial strains producing β-glucosidase

Source of enzyme	Glucose tolerance	Reference
*Jiangella ureilytica* KC603	58% relative activity at 3 M	This study
*Thermotoga naphthophila* RKU-10^ T^	⁓ 40% relative activity at 1 M	[39]
*Exiguobacterium antarcticum* B7	50% relative activity at 1 M	[28]
*Trichoderma harzianum*	30% relative activity at 0.8 M	[39]
*B. subtilis* RA10	70% relative activity at 1 M	[40]
Marine Microbial Metagenome	40% relative activity at 1 M	[41]
*Actinomadura amylolytica* YIM 77502^ T^	AaBGL1 have 40% relative activity at 3 M	[42]
	AaBGL2 have 40% relative activity at 3 M	
Soil metagenomic (Bgl1269)	> 80% relative activity at 3.6 M	[43]

### Kinetics Parameters

**Fig. 7:**
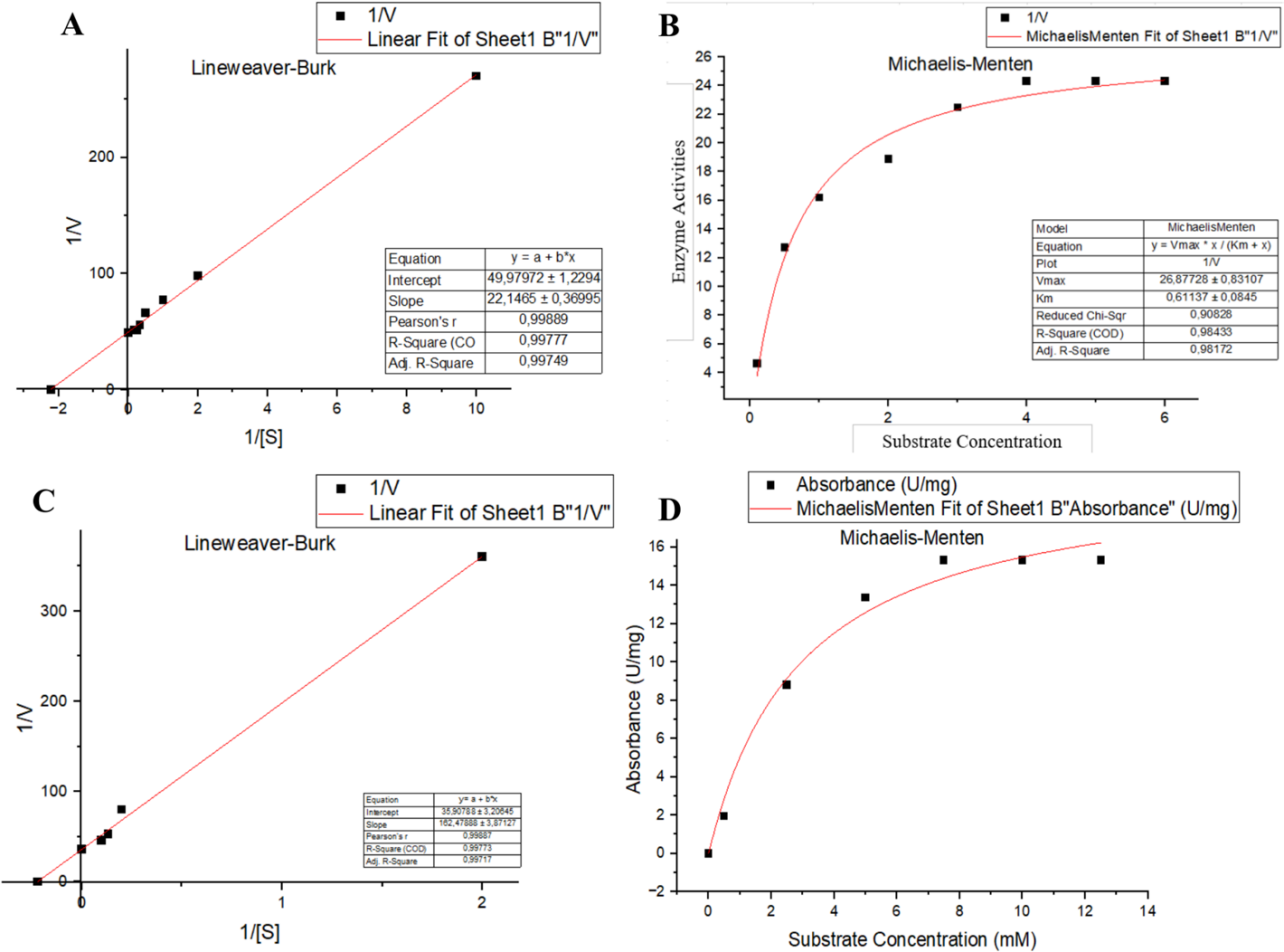
Lineweaver–Burk and Michaelis–Menten plot for hydrolysis of *p*NPG and polydatin. **A** and **B** The Lineweaver–Burk and Michaelis–Menten plot of the *p*NPG-JurBglKC603 reaction. **C** and **D** The graph of Lineweaver–Burk and Michaelis–Menten plot of polydatin-JurBglKC603 reaction

The extinction coefficient of *p*NPG was determined as 12,000 M^−1^ cm^−1^ using colorimetric *p*NP product at 405 nm. This epsilon value was used in further kinetic parameter calculation. The kinetic parameters of the JurBglKC603 were calculated as *K*_m_ = 0.44 mM and *V*_max_ = 26.87 U·mg^−1^ using the Lineweaver—Burk equation (Fig. 7A, B). The turnover number (*k*_cat_) and catalytic efficiency (*k*_cat_/*K*_m_) were calculated as 21.1 s^−1^ and 47,954 M^−1^·s^−1^, respectively, for 0.8 µg of enzyme in 1 mL reaction volume. A detailed comparison of the kinetic parameters of β-glucosidases from different sources against *p*NPG substrate is presented in Table 2.

**Table 2:** Comparison of β-glucosidases of different organisms with *p*NPG substrate

Microorganisms	Physicochemical parameters		Kinetic parameters				Reference
	Optimum pH	Optimum temp (°C)	*K*_m_ (mM)	*V*max (U/mg)	*k*_*cat*_ (s^−1^)	*k*_cat_/*K*_m_ (*M*^−1^ s^−1^)	
*Jiangella ureiltyca KC603*	6	35	0.44	26.87	21.1	47,954	This study
*Alteromonas* sp. L82	7.5	40	1.5	62.5	54.8	36.5	[17]
*Exiguobacterium antarcticum* B7	7	30	1.07	36.7	32.98	30.8	[28]
*Saccharomonospora* sp. NB11	7	40	0.40	5,735.8	5,042.16	12,487.71	[44]
*Micrococcus antarcticus*	-	36.5	5.9	8430	6700	114,000	[45]
*Bifidobacterium longum* H-1	5.5	35–37	0.83	57	-	-	[46]

GOPOD method was used to determine the kinetics of JurBglKC603 for polydatin. In this method, the amount of glucose generated from polydatin de-glycosylation was quantified spectrophotometrically at 540 nm, using a standard glucose concentration [47, 48]. One unit of activity (U) was defined as the amount of enzyme required to produce 1 μmol of reducing sugar per minute under standard assay conditions. A Michaelis–Menten plot was generated using the obtained data, and a Lineweaver–Burk plot was created by plotting the reciprocals of the data points on the *x*- and *y*-axes (Fig. 7C, D). The kinetic parameters of JurBglKC603 for polydatin were determined as follows: 4.6 mM, 20 U·mg^−1^, 17.2 s^−1^, and 3822 M^−1^·s^−1^ for *K*_m_, *V*_max_, *k*_cat_, and *k*_cat_/*K*_m_ values, respectively.

Due to the therapeutic properties of resveratrol, there is growing interest in the enzymatic conversion of polydatin into resveratrol. Several β-glucosidases reported in the literature have high activity in the enzymatic conversion of polydatin to resveratrol [49]. The catalytic properties of the JurBglKC603 against polydatin were compared with those of other β-glucosidases found in the literature (Table 3). The *k*_cat_ and *k*_cat_/*K*_m_ values of JurBglKC603 suggest that it efficiently hydrolyzes polydatin, indicating its potential as an enzyme for polydatin hydrolysis.

**Table 3:** Comparison of kinetics of JurBglKC603 for polydatin with other β-glucosidases

Organizm	*K*_m_ (mM)	*k*_cat_ (s^−1^)	*k*_cat_/*K*_m_ (M^−1^·s^−1^)	Reference
*Jiangella ureiltyca KC603*	*4.6*	17.2	3,822	This study
*Lactobacillus kimchi*	0.20	1.29	6,450	[50]
*Aspergillus oryzae*	0.74	NR	NR	[51]
*Anoxybacillus ayderensis* A9	5.5	18.28	*3,270*	[52]

The turnover numbers of JurBglKC603 against the commercial *p*NPG substrate and polydatin as a natural substrate were both approximately 20 s^−1^. On the other hand, the catalytic efficiency of JurBglKC603 against polydatin was ten times lower than that of *p*NPG, indicating that its affinity towards polydatin was ten times lower. This result is expected, as it is well known that enzymes generally exhibit much lower catalytic activity towards natural substrates.

### Analysis of the Hydrolysis of Polydatin to Resveratrol by TLC and DNS Methods

**Fig. 8:**
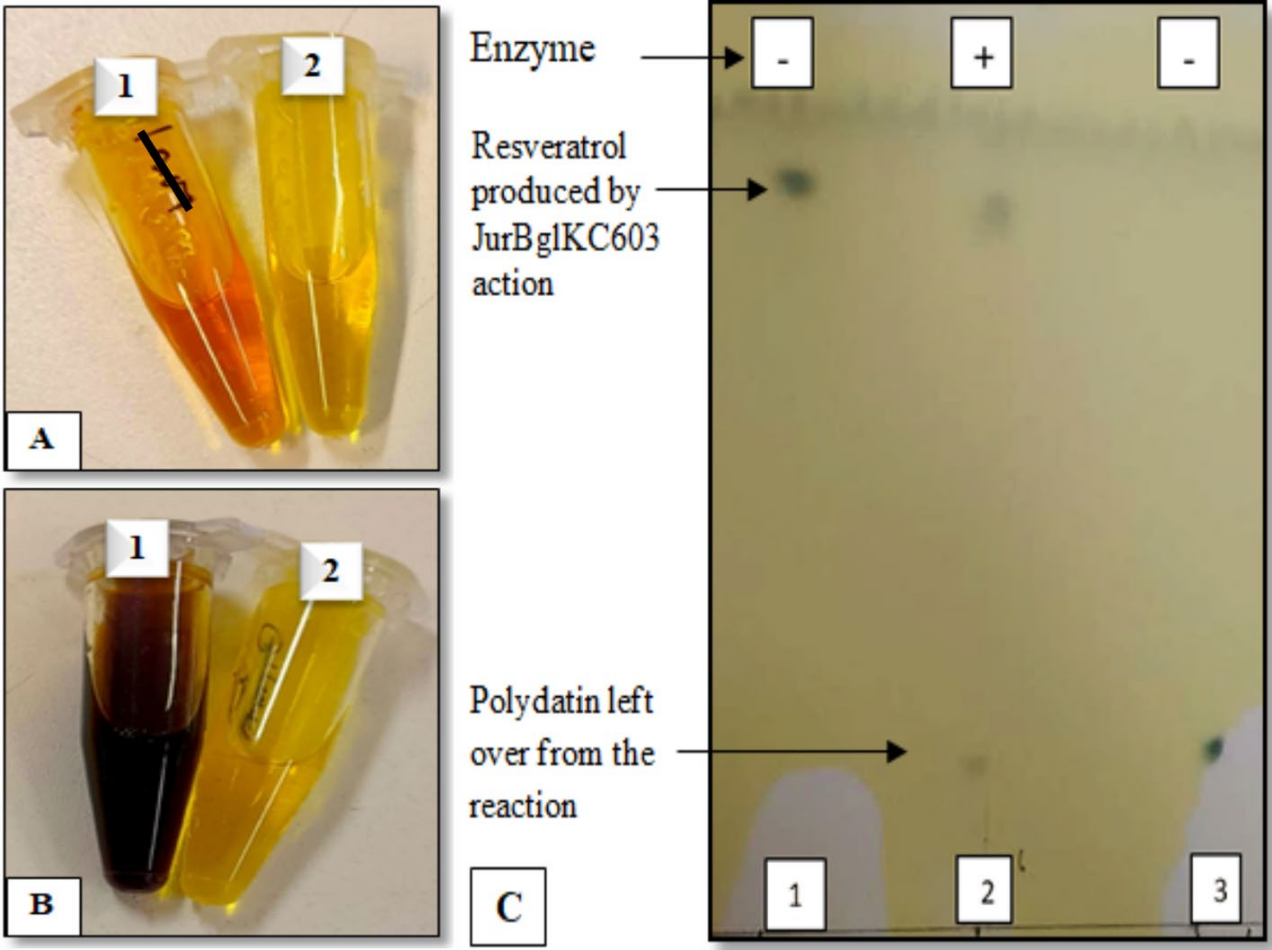
The results of the analysis of polydatin hydrolysis to resveratrol by JurBglKC603 using Thin Layer Chromatography and DNS Assay: **A** (1) the DNS test image capturing the reaction between JurBglKC603 and 0.1 mM polydatin substrate, **A** (2) polydatin as a control (0.1 mM), **B** (1) the DNS test image capturing the reaction between JurBglKC603 and 1 mM polydatin substrate, **B** (2) polydatin as a control (1 mM), and **C** TLC analysis; **C** (1) resveratrol as a control, **C** (2) reaction product of 1 mM polydatin with JurBglKC603, and **C** (3) polydatin (glycosylated) substrate as a control

To confirm the deglycosylation of polydatin, the products formed after the treatment of polydatin with JurBglKC603 were analyzed using TLC and DNS methods. The TLC analysis revealed the presence of resveratrol and non-deglycosylated polydatin following treatment with JurBglKC603 (Fig. 8). This result demonstrates that JurBglKC603 deglycosylates polydatin, leading to the formation of resveratrol.

The DNS analysis quantifies the amount of glucose produced. When 0.1 mM polydatin was used, the measured glucose concentration was 0.46 mg/mL. The glucose formation following the treatment of polydatin with JurBglKC603 confirms the de-glycolisation of polydatin and the release of resveratrol. Since resveratrol has broader applications than polydatin in many areas such as pharmacology, cosmetics, and food, the polydatin conversion capacity of JurBglKC603 to resveratrol is a beneficial property in industrial applications.

### Homology Modeling and Molecular Docking

**Fig. 9:**
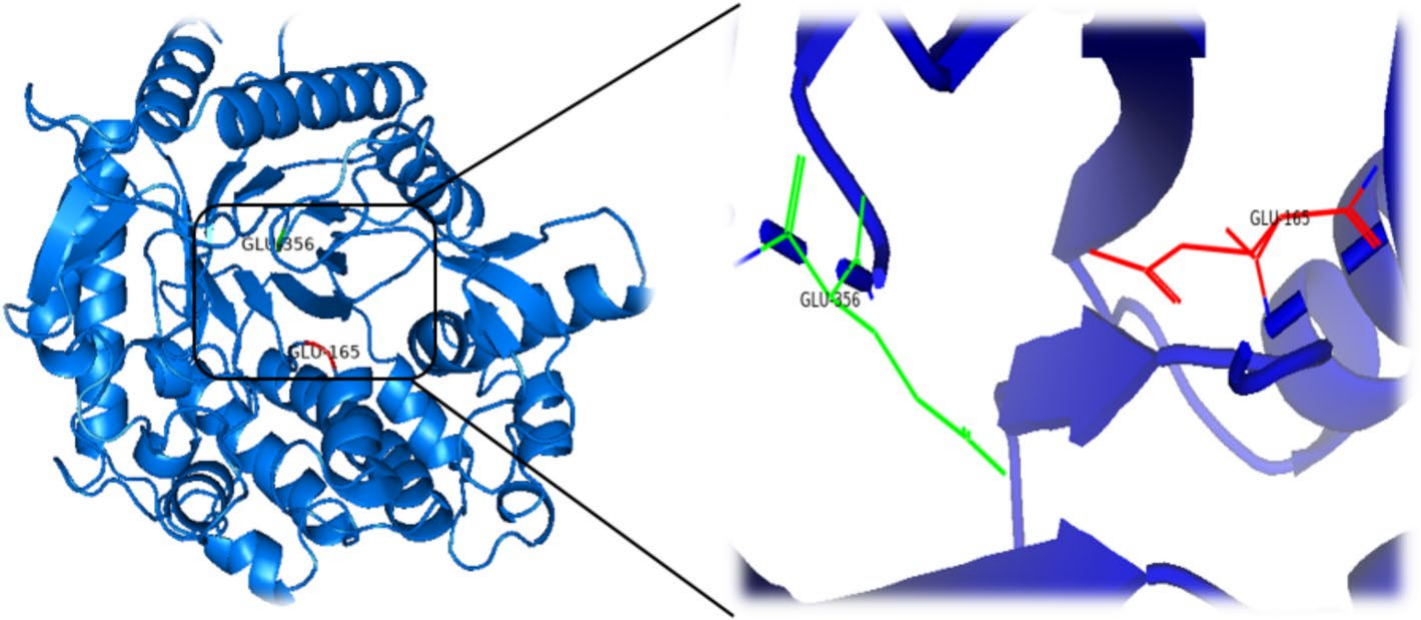
The figure illustrates the modeled 3D structure of JurBglKC603. The predicted nucleophile and acid/base catalytic active site residues of JurBglKC603, namely Glu 165 and Glu 356, are denoted in green and red, respectively. The red-highlighted region corresponds to the protein donor residue, while the green-highlighted region represents the nucleophilic residue. This image was created using PyMOL

**Fig. 10:**
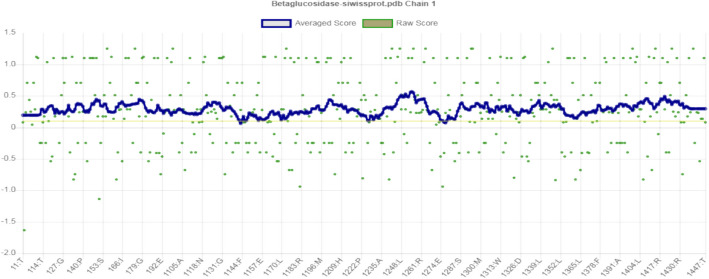
The quality of the protein fold model was assessed using VERIFY3D, revealing that 99.1% of the residues achieved an averaged 3D-1D score of > = 0.1, surpassing the 80% threshold for expected residues

The amino acid sequences of β-glucosidases were submitted to SWISS-MODEL for the construction of 3D structures. The crystal structure of *Kribbella flavida* DSM 17836 (PDB ID: 6M6L) served as a template for the modeling the three-dimensional structure of JurBglKC603 using the SWISS-Model homology modular system (Fig. 9). The root mean square deviation (RMSD) of the 3D structure of JurBglKC603 was calculated as 1.034. The VERIFY3D program was also employed to evaluate the accuracy and quality of the created model (Fig. 10).

The overall quality factor determined by ERRAT was 96.11%, indicating a well-constructed model structure. An ERRAT result exceeding 95% suggests that the model is of high quality and resolution. For models with lower resolutions, the average overall quality factor should be approximately 91%. The model, created based on the results of these analyses, was found to be of good quality.

**Fig. 11:**
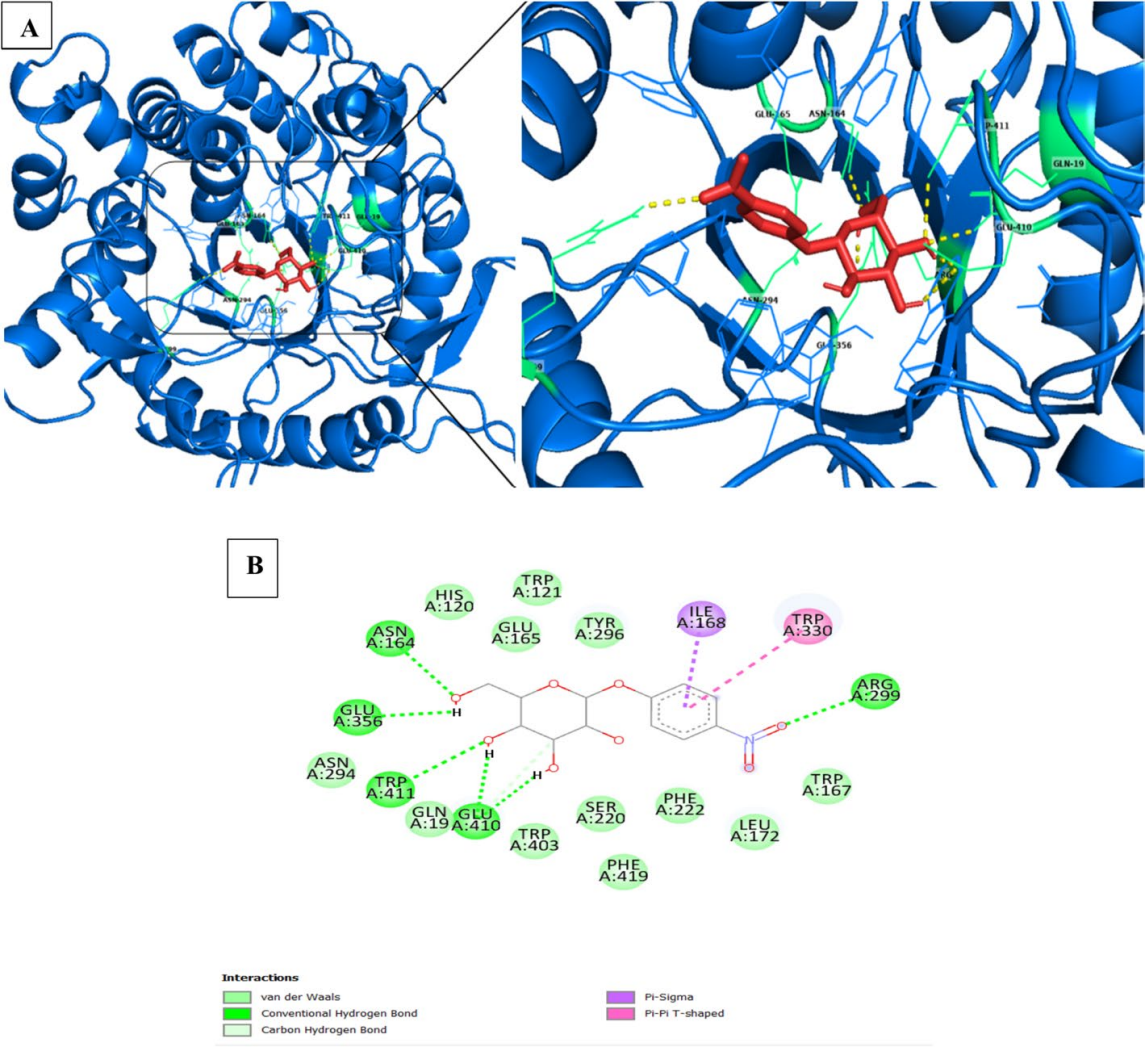
**A** and **B** A cartoon representation of the docked complex involving *p*NPG and JurBglKC603. In **A**, *p*NPG is depicted with red-colored carbon atoms, presented as sticks, in a 3-dimensional (3D) representation. The interacting residues with *p*NPG are shown in green sticks. **B** A 2D image of the docked complex, highlighting residues involved in the hydrolysis of the substrate. AutoDock 4.2 software was used for creating the images

When the *p*NPG substrate is introduced into JurBglKC603, amino acids Arg299, Glu410, Trp411, Glu356, and Asn164 participate in the catalytic mechanism through hydrogen bond interactions with the ligand. These interactions are further supported by non-covalent (hydrophobic) interactions involving Trp121, His120, Glu165, Tyr 296, Asn294, Gln19, Trp403, Ser220, Phe419, Phe222, Leu172, and Trp167 (refer to Fig. 11). Two GH1 residues, Glu165 and Glu356, conserved at these sites, act as catalytic acid/base and nucleophile. These residues are highly conserved among GH1 Bgls and are crucial for enzyme functionality (Fig. 11).

The highly conserved residues in GH1 β-glucosidases play a crucial role in the enzyme’s tolerance to glucose. GH1 β-glucosidases typically exhibit a classical (β/α)8-TIM barrel fold, featuring a cleft-like active site and a variable outer opening, which are effective in processing glucose derivatives of varying lengths [53, 54]. Studies have shown that the deep and narrow substrate channel of GH1 β-glucosidases limits glucose access to the active site, promoting transglycosylation and preventing competitive inhibition [55]. Furthermore, it has been proposed that glucose-tolerant β-glucosidases may contain secondary glucose binding sites within the tunnel, which makes the structural feature crucial for both glucose tolerance and substrate specificity [35].

**Fig. 12:**
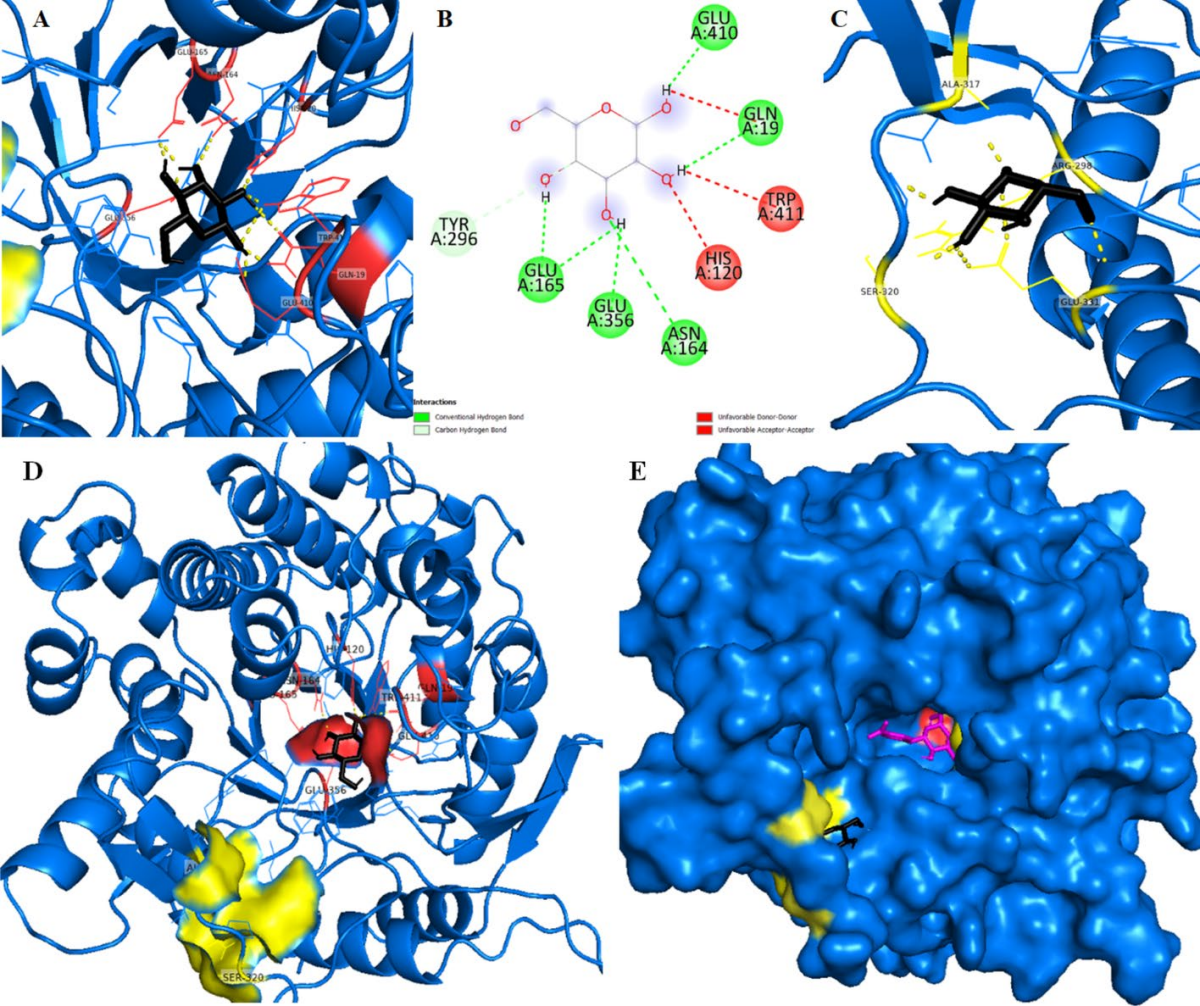
**A**, **B**, **C**, and **D** Created using AutoDock 4.2 software, illustrate a cartoon representation of the docked complex involving Glucose and JurBglKC603. In **A**, **C**, and **D**, glucose is depicted with black-colored carbon atoms in a 3-dimensional (3D) format. In **A**, the region where glucose first interacts is highlighted in red. **B** A 2D depiction of the docked complex, emphasizing residues involved in substrate hydrolysis. **C** The second binding site of glucose, shown in yellow. **D** Both the first binding site of glucose in red and the second binding site in yellow within the 3D structure of β-glucosidase. **E** The regions where Glucose (colored black) and *p*NPG substrate (colored magenta) bind, along with a cartoon representation of the active site (colored red)

To investigate this hypothesis, we initially examined the potential for glucose binding to a hypothetical secondary binding site using the Autodock 4.2 docking program. Analysis revealed the possibility of a secondary binding site for glucose (Fig. 12). It was observed that glucose initially positioned itself near the active site before binding to an adjacent area outside of it. The region where the glucose first settles could potentially serve as a secondary binding site.

The response of GH1 β-glucosidases to glucose depends on the relative binding preferences of glucose to different sites [55]. Inhibition occurs when glucose binds preferentially to the active site, whereas tolerance is observed when it prefers to bind to other sites. In JurBlgKC603, glucose does not directly bind to the active site, instead it binds to conserved regions surrounding the active site. Among these regions, Tyr296 and Trp403 exhibit hydrophobic interactions with glucose, while Gln19, Trp121, Glu410, and Trp411 contribute to glucose binding through weak interactions. However, the importance of glucose binding preference to the second binding site is still not fully understood. While the mechanism of β-glucosidase inhibitions by glucose remains unclear, Molecular Docking studies provide insight into the tolerance of JurBglKC603 to glucose.

**Fig. 13:**
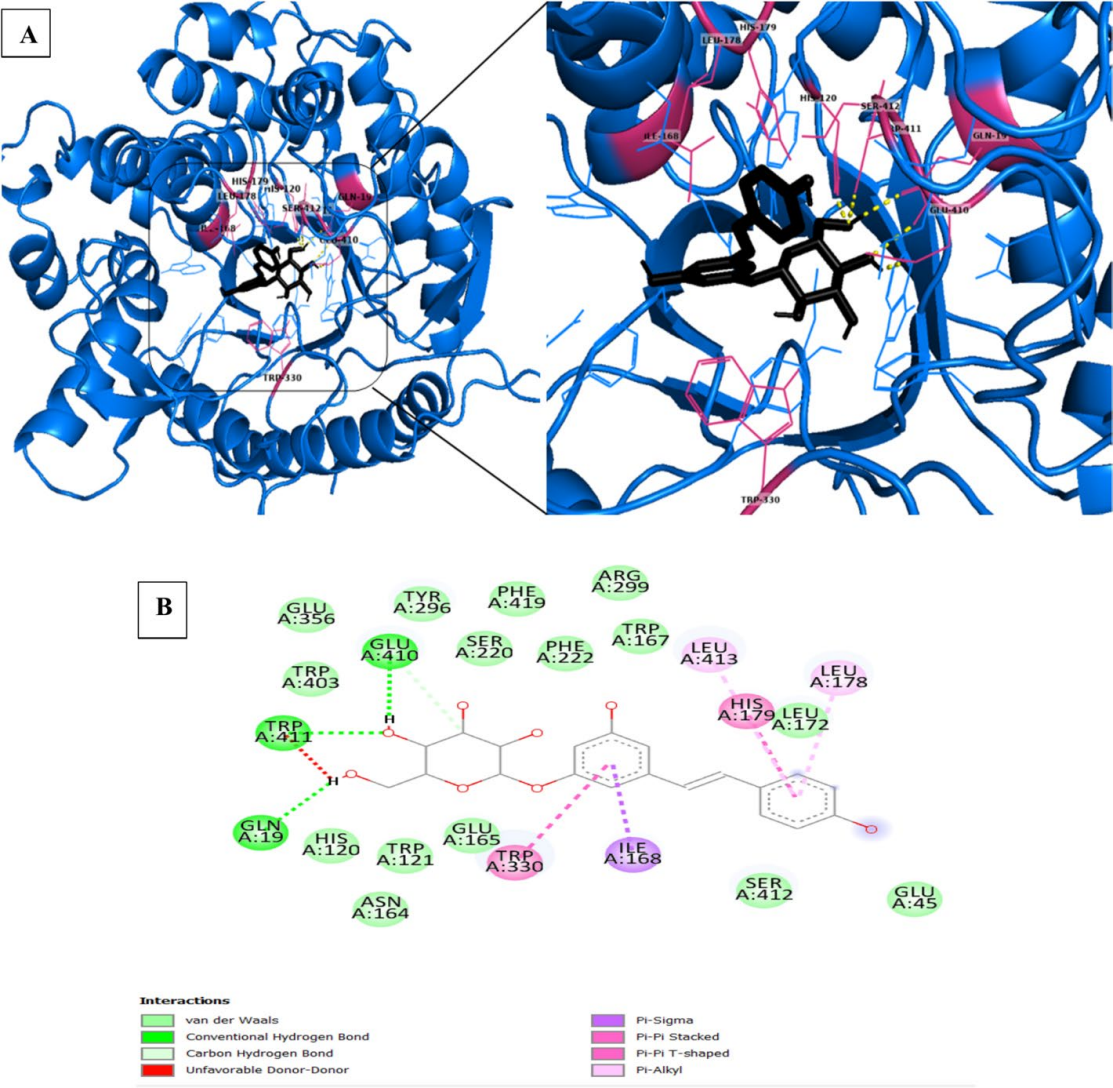
**A** and **B** A cartoon representation of the docked complex involving polydatin and JurBglKC603. In **A**, polydatin is illustrated with black carbon atoms, presented as sticks, in a 3-dimensional (3D) representation. The interacting residues with polydatin are depicted as magenta-colored sticks. **B** A 2D image of the docked complex, highlighting residues involved in the hydrolysis of the substrate. AutoDock 4.2 software was used for docking analyses

Similarly, when polydatin is introduced into JurBglKC603, amino acids Glu410, Trp411, and Gln19 participate in the catalytic mechanism through hydrogen bond interactions. These interactions are complemented by non-covalent (hydrophobic) interactions involving Trp121, His120, Glu165, Tyr296, Asn164, Glu356, Trp403, Ser220, Phe419, Phe222, Leu172, Glu45, Arg299, Ser412, and Trp167 (Fig. 13).

The enzyme’s specific activity is correlated to its efficiency, which is influenced by the precise orientation of the substrate at the enzyme’s active site and its adjacency to catalytic residues [54]. This orientation is connected to the geometry of the active region, determined by the residues constituting the active region and those surrounding it. Within the hydrolytic mechanism of β-glucosidases, two crucial amino acid residues (Glu165 and Glu356) in the active site—a nucleophile and a proton donor—play pivotal roles. Hydrogen bonds and hydrophobic contacts stabilize the enzyme–substrate complex in β-glucosidases, forming interactions between the substrate and side chains of residues located at the glycone (subsite − 1) and aglycone (subsite + 1) binding sites [56]. Along with the highly conserved catalytic residues, other conserved amino acids in the active site of β-glucosidases form direct hydrogen bonds with the glycosyl moiety in the − 1 subsite.

**Fig. 14:**
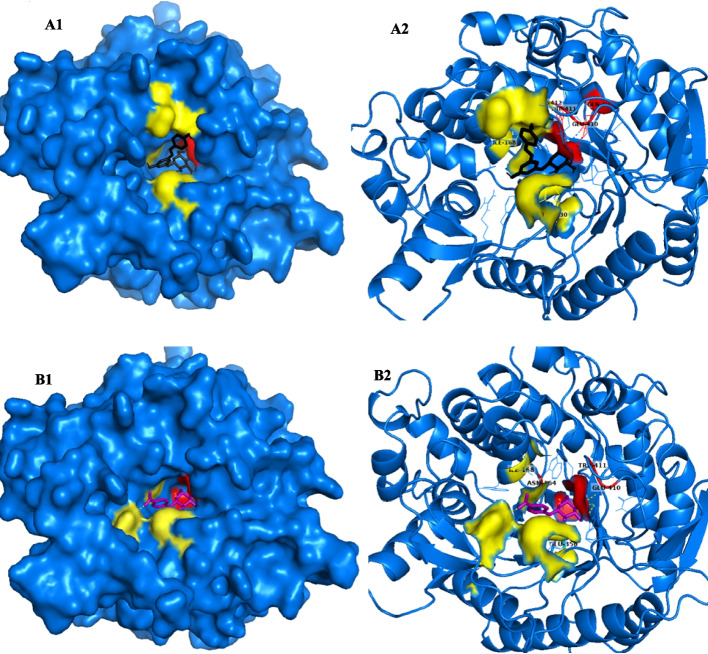
**A** and **B** A cartoon representation of the complex formed between polydatin (**A**1–2) and *p*NPG (**B**1–2) substrates docked with β-glucosidase. The surface representation of JurBglKC603 illustrates the positions of the − 1 (depicted in red) and + 1 (depicted in yellow) subsites within the JurBglKC603 structure. In **A**, the blue molecule represents polydatin, while in **B**, the purple molecule represents the *p*NPG substrate. These images were created using PyMol

The interactions between JurBglKC603 and polydatin and *p*NPG substrates were individually examined using PyMol. In the case of polydatin, amino acids in the regions 19, 410, and 411 formed hydrogen bonds with the glucone regions (subsite − 1) of the polydatin molecule, while amino acids in the regions 330, 168, 178, 179, and 413 interacted with the aglycone regions (subsite + 1), facilitating hydrolysis of the molecule (Fig. 14) [47]. Similarly, during the hydrolysis of the *p*NPG substrate, amino acids in regions 164, 356, 410, and 411 interacted with the glucose moiety of the substrate, while residues in regions 330, 168, and 299 interacted with the aglycone portion of the substrate. Additionally, amino acid residues 120, 121, 165, 296, 356, 403, 412, and 420 contributed to substrate delocalization by forming various bonds, such as carbon-hydrogen and van der Waals, with the substrate [57].

This molecular arrangement provided insights into the amino acid residues involved in substrate hydrolysis within the active site of JurBglKC603 and their crucial roles in enzyme activity.

## Conclusions

A highly active β-glucosidase, JurBglKC603, derived from *Jiangella ureilytica* KC603, was successfully cloned and expressed in *E. coli* BL21 (DE3) cells. The enzyme retained its activity against most of the chemicals analyzed and showed high tolerance to glucose, which is ideal for use in biotechnological applications. JurBglKC603 was active on polydatin and was able to de-glycosylate it to resveratrol. The molecular interaction between polydatin and the modeled JurBglKC603 revealed the presence of conserved residues participating in the enzymatic reaction.

## Data Availability

The data supporting the findings of this study are available upon request.
